# Shaping national rare diseases definition in Saudi Arabia: outcome from health ecosystem multisectoral workshop

**DOI:** 10.3389/fphar.2025.1595967

**Published:** 2025-07-17

**Authors:** Ghada Mohammed Abozaid, Hussain Abdulrahman Al-Omar, Abdulaziz Alrabiah, Hiba Alomary, Amy Jayne McKnight

**Affiliations:** ^1^ Centre for Public Health, Institute of Clinical Sciences B, Royal Victoria Hospital, Dentistry and Biomedical Sciences, Queen’s University Belfast School of Medicine, Belfast, United Kingdom; ^2^ Pharmacy Practice Department, College of Pharmacy, Princess Nourah Bint Abdulrahman University, Riyadh, Saudi Arabia; ^3^ Department of Clinical Pharmacy, College of Pharmacy, King Saud University, Riyadh, Saudi Arabia; ^4^ General Directorate of National Health Policies and Economics, Saudi Health Council, Riyadh, Saudi Arabia; ^5^ Department of Applied Linguistics, College of Languages, Princess Nourah Bint Abdulrahman University, Riyadh, Saudi Arabia

**Keywords:** rare diseases, policy, criteria, definition, Saudi Arabia, reimbursement, regulatory

## Abstract

**Introduction:**

Rare diseases are characterized by low prevalence, a profound impact on patients, and significant challenges in accessing treatment. In Saudi Arabia, the absence of an official definition hampers policymaking, resource allocation, and orphan drugs accessibility. This study aimed to address this gap by proposing a definition of rare diseases using inputs and views from a Saudi multi-stakeholder workshop.

**Methods:**

A 1-day workshop was hosted in collaboration with the Saudi Health Council in Riyadh on 5 June 2023, involving 59 participants from various sectors, including clinicians, policymakers, patient advocates, and industry professionals. Using the Vevox^®^ platform, participants engaged in structured activities comprising a demographic survey and 10 interactive voting sessions. Preferred qualitative and quantitative criteria for defining rare diseases were identified and analyzed using descriptive statistics and thematic and content analysis of open-ended questions.

**Results:**

The findings indicated a strong preference (96%) for an integrated definition combining qualitative and quantitative criteria. Key qualitative terms such as “Disease” (62%), “Serious” (53%), and “Disorder” (51%) were favored for their clarity and broad applicability. “Genetic” etiology was preferred by 91% of participants, citing its relevance and data availability. Patient-centered criteria, including “life-threatening” (73%) and “considerable reduction in quality of life” (91%), were emphasized. Economic and resource-focused considerations, such as “Lack of Resources” and “Combined Efforts to Prevent” (each at 60%), reflected key unmet needs in the current healthcare landscape. Among quantitative criteria, “Prevalence” (82%) emerged as the most accepted, aligned with international practices, although opinions were mixed on the inclusion of population size thresholds, underscoring the definitional complexity of defining RDs with both precision and flexibility.

**Discussion:**

This study provides a foundational basis and scientific root for defining rare diseases in the Saudi context, addressing key gaps in healthcare policies. By integrating evidence-based, patient-centered, and resource-oriented criteria, the proposed definition supports equitable access, improved patient care, and sustainable innovation, in alignment with Saudi Arabia’s Vision 2030 goals. Further refinement with broader stakeholder inputs is essential for the successful integration of Saudi healthcare policies.

## 1 Introduction

Rare diseases (RDs) are a heterogeneous group of disorders that collectively impact more than 450 million individuals worldwide (Repetto and Rebolledo-Jaramillo, 2020; Abozaid et al., 2025a). Over 7,000 RDs have been identified (Haendel et al., 2020; Kaywanga et al., 2022). The genesis of RDs involves several unknown and environmental factors, and more than 80% of them have genetic origins (Gupta et al., 2023). This poses unique challenges for patients, caregivers, healthcare systems, policymakers, and society.

The definition of RDs varies globally and depends on factors, such as healthcare infrastructure, population size, and socio-economic priorities (Chung et al., 2022). Although there is no universally accepted definition (Ma et al., 2013), RDs are characterized by conditions affecting a small population. Prevalence thresholds often guide most definitions; for instance, the European Union (EU) defines RDs as conditions affecting fewer than 1 in 2,000 people, whereas the United States applies a threshold of fewer than 200,000 individuals (Balkhi et al., 2023). However, a recent systematic literature review (SLR) (Abozaid et al., 2025a) analyzing 209 definitions from 93 studies underscores the significant heterogeneity in RD definitions worldwide. The study revealed that while prevalence-based criterion remains the dominant approach, variations exist in thresholds across different jurisdictions, ranging from less than 1 in 50,000 to 6.5 in 10,000 (Richter et al., 2015). Additionally, qualitative criteria, such as disease severity, lack of alternative treatments, and chronicity, also play a role in classification.

Country-specific definitions are, therefore, essential as disease frequencies differ by region owing to genetic, environmental, and demographic factors. These disparities highlight the challenges in standardizing a universal definition and underscore the need for a tailored approach that considers regional healthcare capabilities and patient needs. Ultimately, the lack of a harmonized definition affects research, regulatory policies, and access to ODs, further complicating the already limited treatment landscape (Abozaid et al., 2025a; Walkowiak et al., 2024).

The Saudi Arabian (SA) healthcare system has made significant advancements in recent decades, establishing the country as a regional leader in addressing complex health challenges (Alasiri and Mohammed, 2022). These achievements include remarkable progress in combating prevalent chronic and infectious diseases, driven by robust government initiatives and the development of a comprehensive healthcare infrastructure (Almalki et al., 2011). However, despite this progress, RDs have remained under-recognized within SA healthcare priorities, partially due to their perceived low prevalence, absence of a nationally accepted definition, and complexity of their diagnosis and treatment using orphan drugs (ODs). In addition, the lack of a centralized registry (Cutillo et al., 2017) or database impedes attempts to accurately determine the distribution and prevalence of RDs in SA. This gap limits the ability to identify patients with RDs, allocate resources efficiently, and establish policies that serve the unique needs (Adachi et al., 2023) of these patients.

Furthermore, the lack of a Saudi definition of RDs has far-reaching implications. For instance, the approval process for ODs experiences a significant delay. This regulatory gap also complicates the prioritization of RD research, funding allocation, and collaboration with international organizations (Chan et al., 2020). In addition, the absence of a standardized definition affects the pricing approach for ODs, leading to inconsistencies in cost assessments and reimbursement decisions. The high cost of ODs, often driven by limited patient populations and high research and development (R&D) expenses, exacerbates affordability challenges, making it difficult for public and private healthcare systems to ensure equitable access. Moreover, it imposes a challenge in integrating RDs within insurance coverage in the private sector and the government’s funding and reimbursement system, leaving many patients with limited access to essential diagnostic tests, procedures, and treatments (Fontrier, 2022).

Building on previously published research (Abozaid et al., 2025a) in collaboration with the Saudi Health Council (SHC), which is a regulatory organization dedicated to supervising and enhancing the health sector in SA. It is essential for formulating health policies, enhancing communication among various health agencies, and executing plans to advance public health and well-being, thereby ensuring optimal healthcare standards (Abozaid et al., 2025b). We organized a multi-sectoral, multi-stakeholder workshop and aimed to propose a stakeholder-driven national RD definition in SA. The workshop emphasized collaborative decision-making to ensure a scientifically robust and contextually relevant outcome. In line with Saudi Arabia’s Vision 2030, which prioritizes equitable healthcare, innovation, and improved quality of life (GOV.SA, 2023), the proposed definition aims to strengthen regulatory decision-making, research prioritization, and healthcare planning. By addressing a country’s demographic, clinical, and economic needs while aligning with international standards, this initiative will contribute to a more structured and sustainable RD ecosystem.

## 2 Methods

### 2.1 Study design

A 1-day multi-stakeholder workshop was held in Riyadh, SA, on 5 June 2023, facilitated by the SHC. The aim of the workshop was to develop a country-specific, structured, stakeholder-informed national definition of RDs by engaging official representatives from government, semi-government, and private sectors across the Saudi health ecosystem. The workshop began with a presentation outlining the study’s background, aim, objectives, and key findings from the SLR (Abozaid et al., 2025a), which provided the evidentiary foundation for the criteria discussed. To ensure clarity of these criteria’s meaning, a linguistic specialist reviewed and validated them. This was followed by experts’ consultation with senior policy leaders to validate the criteria in terms of feasibility, applicability, and relevance to the Saudi context. The finalized criteria and their definitions (Supplementary Table S1) were then presented to participants to establish a shared understanding, after which a structured vote was conducted to determine the most applicable and acceptable criteria for inclusion in the national RD definition.

### 2.2 Sampling strategy

A purposeful sampling approach was employed to ensure the inclusion of diverse and comprehensive stakeholders relevant to RD policy development. Invitations were extended to all health sectors, representing both local governments and the private sector. This multi-stakeholder included physicians, pharmacists, health economists, regulators, policymakers, supply chain and procurement specialists, pharmaceutical industry official representatives, academics, patient advocates, caregivers, and health insurance company official representatives. The selection aimed to incorporate various perspectives and expertise to facilitate holistic discussions and informed decision-making during the workshop.

### 2.3 Data collection instruments

The workshop utilized Vevox^®^, an audience engagement for polling, quizzes, Q&A, and surveys, enabling real-time interaction. Participants accessed the platform via their mobile devices by navigating to the Vevox. app and entering a session-specific code. Integrated into PowerPoint presentations, Vevox^®^ displays questions on slides while participants view and submit responses instantly.

The platform was used for interactive activities, including an introductory question to set the stage for discussions, structured voting questions to define RDs, and demographic and professional background data collection to contextualize the responses. These questions addressed qualitative and quantitative criteria and were categorized into six themes (Supplementary Table S2).

Additionally, participants were asked to evaluate each criterion in two dimensions: Acceptability, which involved determining the degree to which a criterion would be tolerable and appropriate by all stakeholders, measured as a percentage, and Feasibility, which required participants to rank the likelihood of relevant data being available and accessible in the Saudi context to support a given criterion.

The participant devices and PowerPoint slides simultaneously displayed the compiled response data, offering immediate feedback following each activity. Vevox was selected because of its accessibility, ease of use, and availability under the university’s software license, ensuring an efficient and engaging workshop. The Vevox-implemented questions are presented in (Supplementary Table S3).

### 2.4 Data analysis

All response data were exported from the Vevox^®^ platform and analyzed using Microsoft Excel. Descriptive statistics, including frequencies and percentages, were used to analyze participant responses to all questions. Demographic and professional background data were summarized to illustrate the diversity of the workshop participants.

For the introductory open-ended question, a thematic analysis was conducted following the method outlined by Braun and Clarke (Braun and Clarke, 2006). Two reviewers (GMA and HAA) independently performed the analyses to ensure reliability and minimize bias. This systematic approach involved familiarizing researchers with the data, generating initial codes, and identifying patterns across the responses. These codes were then grouped into overarching themes to capture participant perspectives on decision-making regarding RD definitions. The results are presented in tables/figures illustrating the identified themes and their corresponding codes to provide a clear and structured representation of the findings.

### 2.5 Ethical considerations

The study protocol was evaluated and approved by the Princess Nourah Bint Abdulrahman University (PNU) Institutional Review Board on 18 May 2023. The IRB Registration Number with KACST, KSA is HAP-01-R-059.

## 3 Results

### 3.1 Participants demographic information

A total of 59 participants were included in the study; most were male, accounting for 37 (63%). Demographic information of the participants is presented in (Table 1).

**TABLE 1:** Participant demographic information.

Characteristics	Participants (N)	Percentage
Gender		
Male	37	63
Female	12	20
Age (years)		
25–34	8	14
35–44	19	32
45–54	15	25
≥55	7	12
Educational background		
Physician	25	42
Pharmacist	18	31
Other	8	14

**FIGURE 1:**
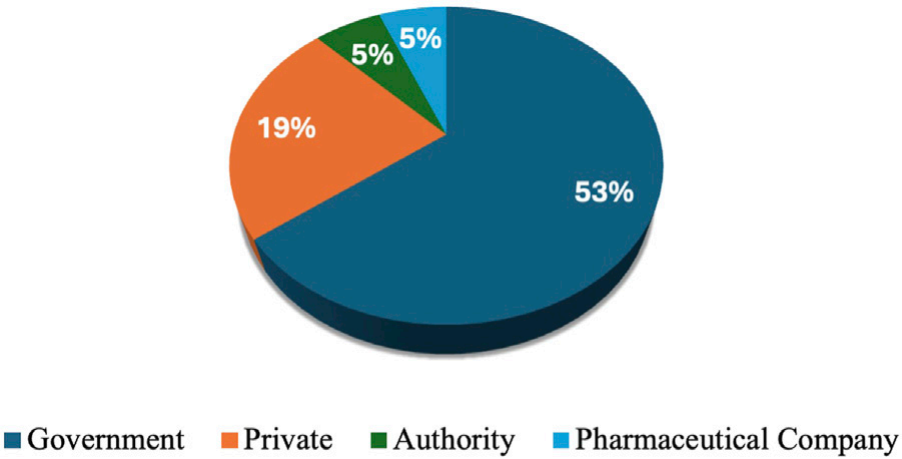
Percentage of the workplace setting nature.

Workshop participants represented diverse and multidisciplinary groups. Among the attendees, 10 (17%) were clinical pharmacists, 11 (19%) were chairs or members of pharmacy and therapeutic committees (PTCs), 12 (20%) were academic professors, and 5 (8%) were policymakers. Additionally, the participants included 5 (8%) key opinion leaders, 2 (3%) official representatives from payer organizations, 4 (7%) regulatory officials, and 5 (8%) researchers affiliated with research agencies. The workshop also featured 8 (14%) experts in clinical guideline development, 6 (10%) health authority officials, and 23 (39%) physicians. Furthermore, the group included one medical insurance representative and 13 (22%) individuals from other health-related disciplines, who collectively contributed valuable perspectives aligned with the workshop’s objectives. More than half (53%) of participants worked in government settings. The private sector accounted for 19% of participants. Both authorities and other workplace settings accounted for a small proportion (5%) (Figure 1).

### 3.2 Criteria to define RD

To understand how stakeholders balance evidence, practicality, and ethics in shaping RD definitions, the introductory question, “What criteria to define RD help you to decide whether to say ‘no’ to something or commit to it?” was designed. This question sought to identify essential factors, such as ethical considerations and societal impact, and assess the applicability and feasibility of the proposed criteria within the healthcare system. The participants provided various criteria that influenced their decision-making processes (Table 2). Notably, 24% of participants did not respond to this question, reflecting that partial engagement with this aspect of the discussion could potentially indicate a lack of clarity.

**TABLE 2:** Participant responses on decision-making criteria for RD definitions.

Theme	Key aspects	Key criteria mentioned
Evidence-Based Decision-Making	Emphasis on scientific rigor, measurable outcomes, and data-driven justifications	• “Evidence-based” • “Scientific evidence” • “Statistics, evidence” • “Clear objectives” • “Facts, numbers, logic, studies, and results” • “Based on the cost-effectiveness analysis of orphan drugs” • “Effectiveness (based on previous patients and literature)” • “Measurable outcomes” • “Supported details”
Patient-Centric Criteria	Focus on patient outcomes, suffering, and the implications of decisions on patient care	• “Impact on patient care, system, and healthcare providers” • “Patient suffering” • “The implication on the patient” • “Clinical benefits” • “Comprehensive care programs including prevention resources” • “Safety and efficacy” • “Outcome” • “Value and outcomes”
Cost and Economic Impact	Assessment of affordability, cost-effectiveness, and financial sustainability	• “Cost impact, valued results” • “Cost, prevalence, outcome” • “Financial impact of expenditures” • “Cost-effectiveness analysis” • “Method of reimbursement” • “Budget impact of orphan drugs” • “Risk benefit”
Safety and Efficacy	Emphasis on the safety profile, efficacy, and reliability of treatments or decisions	• “Safety, efficacy, and cost-effectiveness” • “Harm associated with the decision” • “Efficacy and safety” • “Say no to orphan drugs that have no solid outcomes data” • “Say no to prohibitively expensive medications”
Ethical and Equity Considerations	Focus on fairness, equal access, and alignment with ethical principles	• “Ethical aspects, fairness, ability to provide equally” • “Wisdom behind it” • “Expected commitments and outcomes” • “Just and equitable” • “Clear and specific for better alignment with all stakeholders”
Feasibility and Practicality	Assessments of resource availability, accessibility, and practical implementation of criteria	• “Practically relevant, accessible, easy availability” • “Ability to fulfill this commitment” • “Time required to reach the medication” • “Team members” • “Ease of use and simplicity” • “No medication treatment for this disease”
Quantitative and Qualitative Balance	The balance of numerical thresholds with qualitative insights for comprehensive evaluation	• “Quantitative and qualitative data” • “No fixed number to define a rare disease” • “Logic and balanced decisions” • “Offering win-win situations”
Societal and Long-Term Implications	Broader considerations such as the societal burden, prevention strategies, and long-term impact	• “The burden of the disease (prevalence, economic impact)” • “Short- and long-term impact on society” • “Preventive measures to mitigate future occurrences” • “Direct and indirect economic impacts”

The most significant factors were the evidence of an impact on patient care and society. Cost and expected outcomes also played crucial roles in decision-making, indicating a strong focus on practical and evidence-based criteria. Conversely, several criteria, such as efficacy, logic, and safety, were less frequently cited, suggesting that, although they are still important, they may not be the primary drivers in the decision-making process for many participants.

Regarding the comprehensive Saudi definition of RD, most participants (96%) expressed a preference for using both qualitative and quantitative criteria. Only 4% of the participants favored relying solely on qualitative criteria, indicating a robust agreement for a comprehensive strategy that incorporates both forms of data.

The participants deliberated on the inclusion of population size criteria in the definition of RDs. Most participants (60%) supported the inclusion of a population size criterion, citing its ability to provide objective criteria, avoid uncertainty, assist in disease classification, clarify definitions in publications, estimate burden, and aid in planning resource allocation, budgets, and services. These justifications reflect the importance of measuring and standardizing parameters to enhance clarity and operational efficiency. In contrast, 40% of participants opposed the inclusion of the population size criterion. Their concerns included the lack of reliable local data, the subjectivity of population changes over time, and the risk that numerical thresholds may not adequately reflect the significance of a disease. In addition, they highlighted potential biases, the exclusion of important conditions, and a preference for a dynamic and generalizable definition.

### 3.3 Stakeholder preferences for qualitative criteria in defining RDs

**FIGURE 2:**
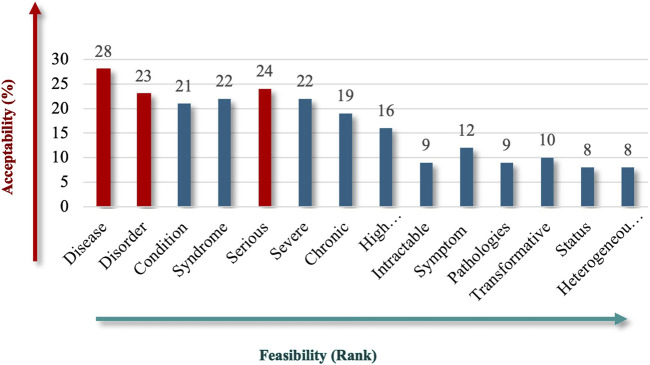
“Nature” theme.

Participants were asked to identify the most acceptable and feasible qualitative criteria for inclusion in the Saudi definition of RD, emphasizing preferences for clear, actionable, and universally recognized terms. “Disease” (62%), followed by “Serious” (53%), and “Disorder” (51%) stood out as the top criteria for the nature of RDs (Figure 2), reflecting familiarity, broad medical applicability, and the fact that these criteria cover a variety of clinical presentations. Criteria like “Syndrome,” “Severe,” and “Condition” also received substantial support, each with acceptability rates above 40%, but scored lower in terms of feasibility, possibly because they are seen as somewhat subjective or context dependent. This result indicates participant interest in conveying the severity and complexity of RDs.

**FIGURE 3:**
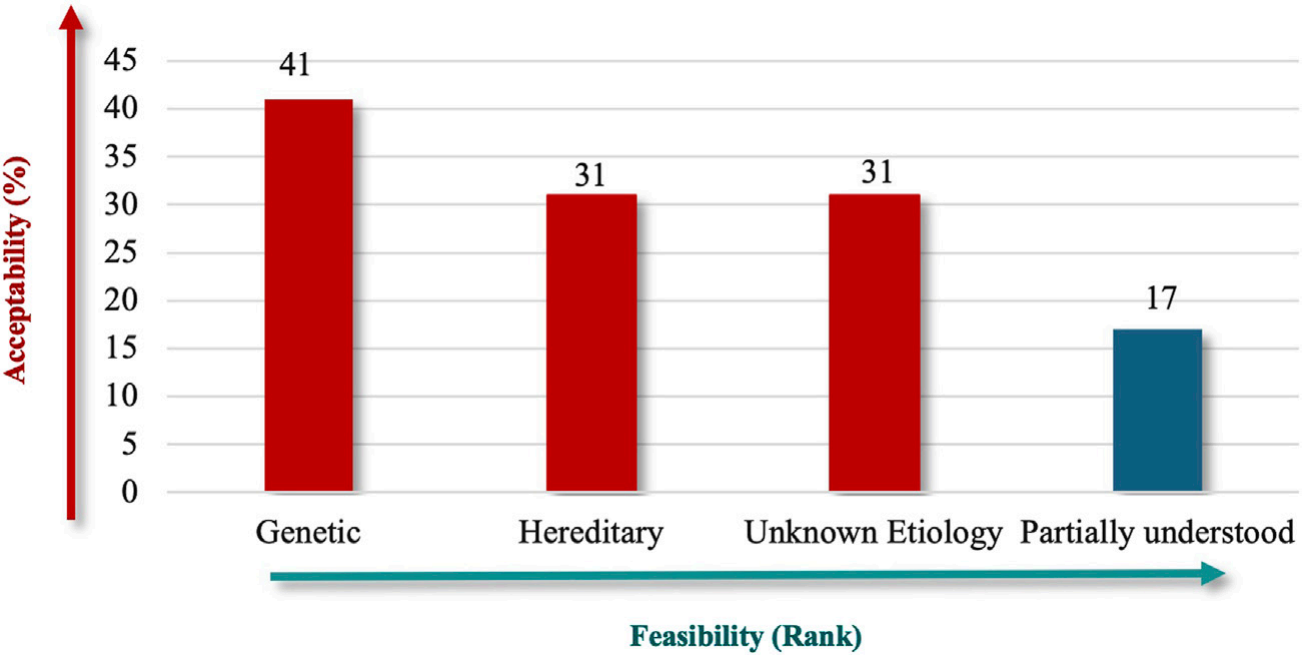
“Etiology” theme.

Due to its established role in RDs and the availability of supporting data, “Genetic” (91%) received high priority in terms of etiology, followed by “Unknown Etiology” and “Hereditary” (both 69%) (Figure 3). The lower ranking of “Partially Understood” suggests that participants prefer criteria that are definitive, which could help make the Saudi RD definition more robust and easier to apply in practice.

**FIGURE 4:**
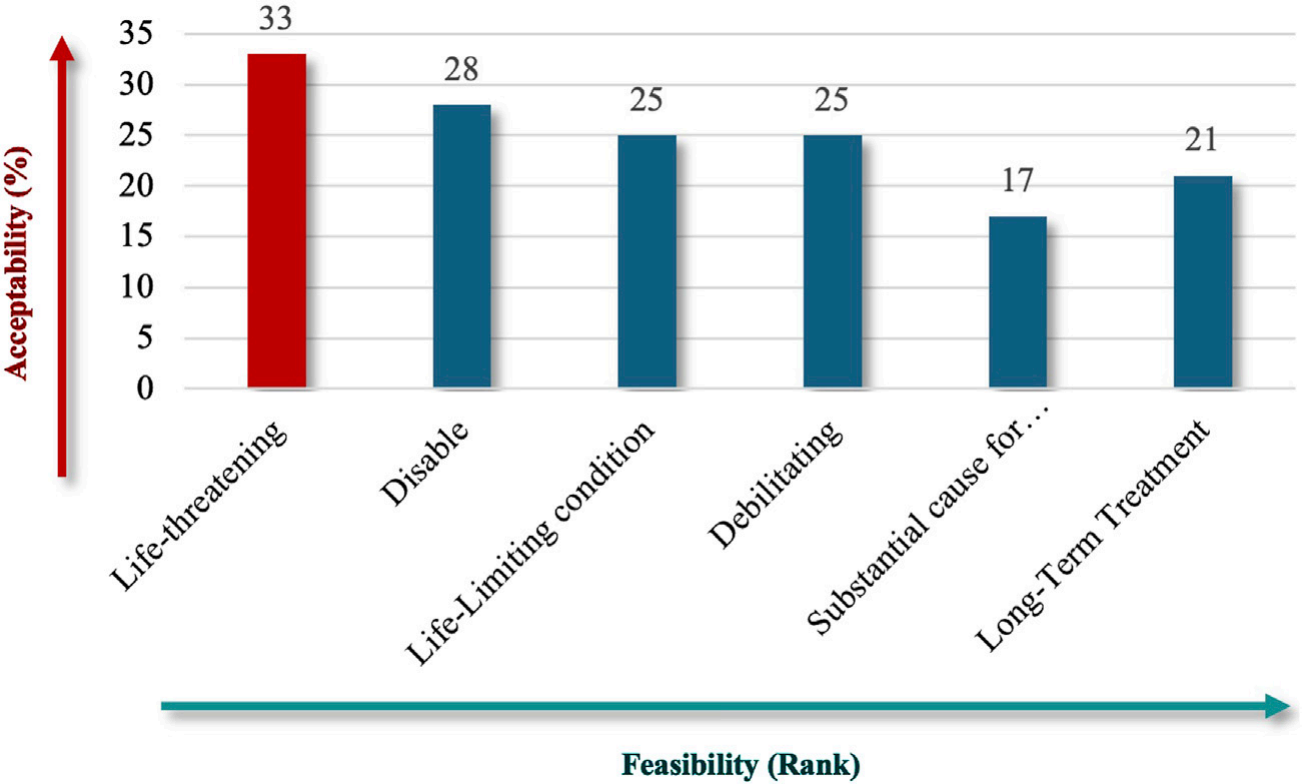
“Disease nature affecting the patient” theme.

**FIGURE 5:**
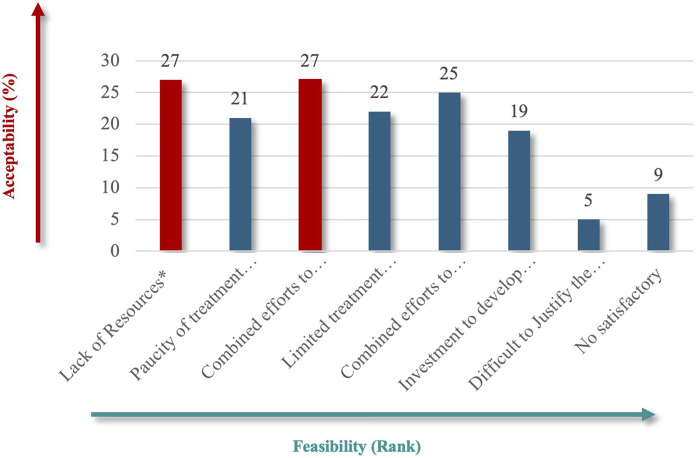
“Unmet needs” theme.

For patient impact, “life-threatening” (73%) emerged as the most critical criterion (Figure 4), emphasizing the severe and immediate health implications of RDs. Under unmet needs, respondents equally favored “Lack of Resources” and “Combined Efforts to Prevent” (60%) (Figure 5), highlighting the importance of addressing resource gaps and promoting coordinated actions to mitigate RD challenges.

**FIGURE 6:**
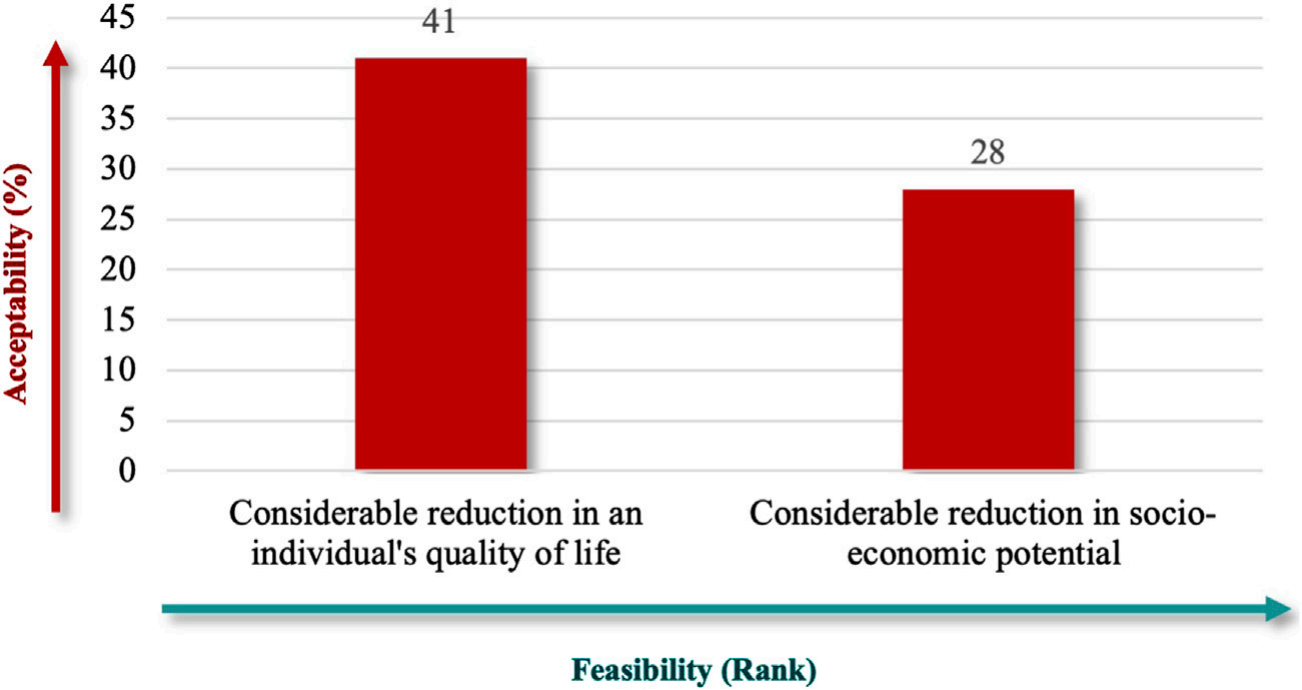
“Disease nature affecting the patient’s society” theme.

**FIGURE 7:**
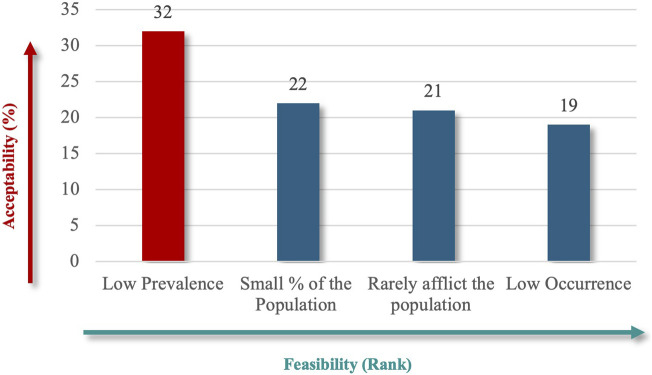
“Population characteristics” theme.

“Considerable Reduction in an Individual’s Quality of Life” (91%), followed by “Considerable Reduction in Socio-Economic Potential” (62%) (Figure 6), which emphasize patient-centered outcomes, and financial implications best captured the societal impact. Finally, the criteria specify whether RD definitions should explicitly reflect the number of people affected, using terms like “low” or “near cutoff” to indicate rarity thresholds. The lowest prevalence criteria were the highest-ranked criteria for population characteristics (71%) (Figure 7), aligned with the international standards for defining RDs.

### 3.4 Stakeholder preferences for quantitative criteria in defining RDs

**FIGURE 8:**
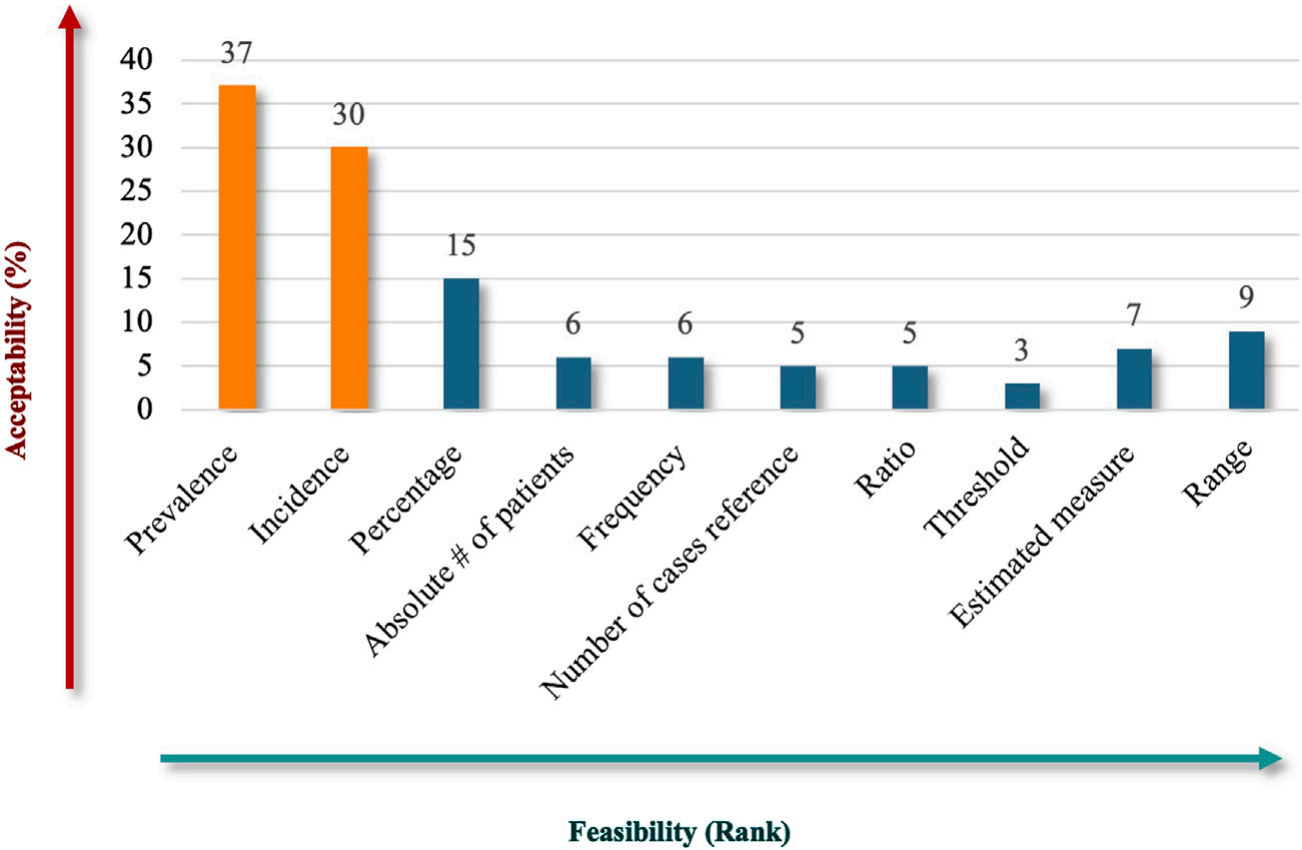
“Measurements” theme.

The results showed that participants preferred to include quantitative criteria in the Saudi definition of RD, proposing various measurements to convey the rarity of the diseases (Figure 8). “Prevalence” emerged as the most acceptable (82%) and feasible descriptor, reflecting a strong stakeholder alignment with its relevance as a core metric for RD definitions, making it a widely recognized standard in healthcare contexts. “Incidence” ranked second in acceptability (67%), indicating its importance in capturing the frequency of new cases and complementing the prevalence in defining RDs. While “percentage” criteria may not be as universal or practical as prevalence and incidence, they still hold relevance in specific contexts. Other terms, like “estimated measure” and “range,” showed limited acceptability but may have niche applications. The “absolute number of patients,” “frequency,” “number of cases referenced,” and “ratio” received limited support, reflecting challenges in their applicability or alignment with stakeholder expectations. The ambiguity or inconsistent application of the “Threshold” criteria probably led to criticism.

## 4 Discussion

Our findings revealed a comprehensive definition that aligns with Saudi healthcare priorities while addressing the unique challenges faced by the healthcare system. Developing a Saudi definition of OD is critical to advance evidence-based policymaking, improve resource allocation, and foster innovation in OD development, all of which are essential components of Vision 2030. To the best of our knowledge, this is one of the first studies worldwide to establish an RD definition based on scientifically sound descriptors, stemming from a SLR of 209 distinct RD definitions across multiple jurisdictions (Abozaid et al., 2025a), structured stakeholder input obtained through a participatory workshop, and validation of quantitative and qualitative criteria specific to SA.

Based on our findings, we propose the following national definition of RDs in SA: A serious and life-threatening disorder with a genetic, hereditary, or unknown etiology that significantly reduces an individual’s quality of life and has a low prevalence in the population, characterized by a lack of available treatment options, substantial medical and socio-economic burden, and requiring specialized interventions. While the proposed definition emphasizes “genetic origin” as a primary etiological criterion, this reflects the prioritization made by the workshop’s official representatives, given the high prevalence of genetic conditions among recognized RDs in SA (Abozaid et al., 2025b). However, not all RDs are of genetic origin, to maintain inclusivity and prevent overly narrow categorization, the final phrasing includes “genetic, hereditary, or unknown etiology.” This broader formulation of definition terminologies ensures that conditions of non-genetic origin, such as certain rare cancers, autoimmune diseases, and rare infectious diseases, are not excluded by default.

A prominent theme was evidence-based decision-making, emphasizing scientific rigor and reliance on clinical trials, statistical comparisons, and measurable outcomes. This aligns with international standards and ensures that healthcare interventions are effective, safe, and supported by robust data (Boeira et al., 2025). Additionally, patient-centric criteria were highly prioritized, with stakeholders underscoring the importance of improving patient care, outcomes, and quality of life. This reflects the ethical obligation to address the complex and underserved needs of patients with RDs, often through comprehensive care programs and prevention strategies.

Economic considerations are central to decision-making, particularly in the Saudi context, where the high costs of ODs are compounded by inconsistent planning and reimbursement policies and strained healthcare budgets. Globally, the life sciences industry spends approximately $5 billion annually on R&D for innovative drugs, with an average of only four new drugs approved for marketing each year over the past decade (Mak et al., 2024), underscoring the substantial investment required for drug development. Our stakeholders emphasized the importance of cost-effectiveness, affordability, and financial sustainability as essential factors aligning with Saudi and global trends. A structured approach to RD definitions that incorporates economic evaluations can facilitate resource allocation and enhance the feasibility of OD development. As suggested in similar studies (Cui and Han, 2015; Lekander and Ylva, 2010; Harris, 2018; Straum, 2018; Antoñanzas et al., 2017), a prevalence-based definition considers factors such as R&D costs, sales revenue required for profitability, and healthcare affordability to establish thresholds that foster innovation and improve access to treatments. For instance, a study in China (Cui and Han, 2015) proposed a prevalence-based definition to guide OD designation, emphasizing the need for specific thresholds to stimulate innovation and improve access to the healthcare system. However, some prevalence figures have been extrapolated, and actual patient numbers often remain uncertain (Song et al., 2017), highlighting the need for reliable data to refine these thresholds further.

This study also identified safety and efficacy as critical criteria, reflecting the cautious approach required for RD policy development, regulatory approval, and healthcare decision-making. Stakeholders emphasized the importance of strong safety profiles and proven efficacy in alignment with national and international standards. Similarly, ethical and equity considerations were integral to the decision-making process and highlighted the need for fairness and equitable access to treatment, addressing the broader societal responsibility of healthcare systems.

Feasibility and practicality have emerged as key themes, often associated with ensuring that decisions are realistic, implementable, and achievable within existing system constraints. However, unlike definitions that merely describe feasibility as the possibility of implementation without empirical validation, a more rigorous perspective incorporates measurable criteria, such as cost-effectiveness (Nicod et al., 2019), resource availability, stakeholder acceptance, and systemic adaptability. Some definitions of feasibility lack a scientific foundation and rely on broad assumptions rather than evidence-based frameworks (Mohammadshahi et al., 2022; Amrein, 2020), making them subjective and inconsistent across different contexts. Without clear evaluation metrics (Zelei et al., 2021), feasibility risks become an abstract and ambiguous concept rather than a structured determinant in decision-making. To address this limitation, feasibility should be assessed using a structured framework that integrates qualitative and quantitative measures to ensure practical applicability and alignment with real-world constraints.

Our findings demonstrate an appreciation of the balance between quantitative and qualitative criteria. While numerical thresholds and statistical metrics offer clarity and precision, qualitative criteria, such as societal impact, ethical considerations, and patient narratives, contribute to depth and context. This dual approach ensures a comprehensive and adaptable definition of RD.

Finally, our participants highlighted the societal and long-term implications of decisions, emphasizing the broader impact on public health, economic burdens, and the need for preventive strategies. This perspective aligns with Saudi Arabia’s Vision 2030 (Program, 2021), which prioritizes sustainable healthcare systems and signaling, incentivizes innovation, and ensures equitable access, all aligning with the country’s ambitious vision, as well as strategies for biotechnology and localization. Criteria addressing these implications, such as “Considerable Reduction in Quality of Life,” received strong support for their relevance and feasibility, reflecting the direct effects of RDs on patients. Socio-economic criteria, such as “Reduction in Socio-Economic Potential,” received moderate support, suggesting they are viewed as secondary to the direct effects on quality of life. These criteria highlight the economic and productivity challenges faced by individuals with RDs, emphasizing their broader social and financial impacts. Despite being secondary, these criteria remain essential in addressing the wider implications of RDs.

In terms of criteria, participants favored terms, such as “Disease,” “Genetic,” and “Life-Threatening,” which scored high in both acceptability and feasibility. These terms provide clarity and align with international practices (Richter et al., 2015; Schieppati et al., 2008). Conversely, terms like “Heterogeneous” and “Partially Understood” were less favored due to their perceived vagueness and limited applicability. Similarly, while population-based criteria, such as prevalence and incidence, were seen as crucial, participants acknowledged the need to integrate these metrics with qualitative aspects to create a comprehensive and balanced definition.

Overall, the findings emphasize a clear preference for established, measurable, and practical criteria, such as prevalence and incidence, while highlighting the challenges associated with less defined or ambiguous metrics. These results underscore the importance of selecting quantitative criteria that balance conceptual clarity, stakeholder alignment, and data availability when defining RDs.

The inclusion of a population size criterion in the Saudi definition of RD received a high vote from most participants, although a minority opposed it. This might reflect the challenge of balancing objective criteria with the flexibility required to address contextual and evolving factors. A strict but flexible approach is required to define RDs. Discussions should weigh the advantages and disadvantages of both perspectives to ensure that the final definition fits the needs of all stakeholders and the local healthcare system.

The term “low prevalence” was included in the definition to align with SA’s evolving RDs landscape and broader health sector transformation (Al-Omar et al., 2025), based on inputs from workshop official representatives. Although fixed numerical values provide quick clarity for payers, policymakers, and regulators, they may become obsolete as healthcare reforms, infrastructure, monitoring systems, and disease registries advance. While “low prevalence” may be challenging to interpret, national health authorities must establish contextual benchmarks by utilizing disease burden modeling, registry data, and ongoing expert consultations to address this challenge. This enables flexibility throughout implementation while maintaining operational consistency and clarity. As national data systems evolve, a fixed numerical value may be defined and periodically revised to correspond with the country’s national priorities.

Regarding the definition, most participants (96%) preferred to use both qualitative and quantitative criteria. However, 4% preferred only the qualitative criteria, indicating broad support for a comprehensive approach that integrates both data types. This outcome highlights the participants’ understanding of the importance of balancing numerical thresholds that define rarity with qualitative considerations, such as societal impact and ethical considerations, to provide a comprehensive and reliable definition of RDs.

These results suggest that RDs must be defined in SA in a structured and flexible manner. The definition should reflect both personal and societal perspectives, incorporating quantitative and qualitative criteria to ensure it is comprehensive and operational. By integrating evidence-based, ethical, patient-centered, and economically feasible criteria, SA can establish an RD definition that can drive innovation, improve patient outcomes, and ensure equitable access to healthcare systems, services, and innovative and breakthrough therapies. A Saudi context-tailored definition should incorporate a prevalence threshold that adapts to changes in drug development costs, sales, and system affordability. The suggested threshold is a starting point, with the final definition requiring granular data on investment returns in the SA, which can support effective policymaking and enhance resource allocation.

This study presents several key strengths stemming from its inclusive design and methodological rigor. First, stakeholder engagement was conducted comprehensively, with invitations extended to the entire Saudi health ecosystem through the SHC, the highest regulatory health authority in SA. Second, the study was grounded in evidence derived from an extensive SLR of RD definitions used across countries, jurisdictions, and regulatory agencies. Third, the involvement of a linguistic specialist enhanced the methodological rigorousness of the study by ensuring terminological precision of the criteria presented for stakeholder voting. Fourth, the participatory workshop, combined with real-time voting tools, facilitated the rapid and transparent collection of responses from official stakeholders’ representatives across diverse professional backgrounds and open-ended feedback. Finally, the used approach, with its thick description, supports method transferability, particularly for global health systems that either lack country-specific, developed, or adopted RDs definitions from other nations or regions.

On the other hand, this study has some limitations that should be acknowledged. The perspectives of certain stakeholder groups may have disproportionately influenced the outcomes, particularly due to the underrepresentation of patients and patient advocates. Although patients were invited, none participated directly; however, input was captured through a healthcare professional who also served as a patient representative. The single-day workshop format may have limited the depth of discussion, constraining extensive deliberation on certain criteria. Additionally, the qualitative criteria were assessed through self-reported feedback, which may reflect individual biases. To overcome these limitations, future studies should consider increasing the sample size, conducting multi-day research, and ensuring broader representation, particularly of patients and caregivers. Incorporating comprehensive national data will also help refine and validate the proposed definitions of RDs.

## 5 Conclusion

This study marks a critical step in establishing a Saudi definition of RDs, which can address a significant gap in healthcare policy and practice and consequently impact the system. Through a comprehensive, stakeholder-driven workshop, the findings highlight the importance of integrating both qualitative and quantitative criteria that are locally feasible and applicable. This approach balances objective metrics, such as prevalence and incidence, with qualitative aspects, including societal impact, ethical considerations, and patient outcomes, and provides a scientific foundation for the definition. By addressing societal, ethical, and economic considerations, the proposed definition promotes equitable access to care, effective resource allocation, and the development of innovative therapies and healthcare solutions. Future studies should refine the definition and assess its impact using broader inputs.

## Data Availability

The original contributions presented in the study are included in the article/Supplementary Material, further inquiries can be directed to the corresponding author.

## Abbreviations

EU, European Union; FDA, Food and Drug Administration; OD, Orphan Drugs; PNU, Princess Nourah Bint Abdulrahman University; QUB, Queen’s University Belfast; RD, Rare disease; SA, Saudi Arabia; SFDA, Saudi Food and Drug Authority.

## References

[B1] Abozaid G. M. Al-Omar H. A. Alrabiah A. AlMuaither A. McKnight A. J. (2025b). Towards a sustainable rare disease and orphan drug ecosystem in Saudi Arabia: policy insights from a multi-stakeholder workshop. Front. Pharmacol. 16, 1583477. 10.3389/fphar.2025.1583477 40376269 PMC12078156

[B2] Abozaid G. M. Kerr K. Alomary H. Al-Omar H. A. McKnight A. (2025a). Global insight into rare disease and orphan drug definitions: a systematic literature review. BMJ Open 15 (1), e086527. 10.1136/bmjopen-2024-086527 PMC11784410 39863413

[B3] Adachi T. El-Hattab A. W. Jain R. Nogales Crespo K. A. Quirland Lazo C. I. Scarpa M. (2023). Enhancing equitable access to rare disease diagnosis and treatment around the world: a review of evidence, policies, and challenges. Int. J. Environ. Res. public health 20 (6), 4732. 10.3390/ijerph20064732 36981643 PMC10049067

[B4] Alasiri A. A. Mohammed V. (2022). Healthcare transformation in Saudi Arabia: an overview since the launch of vision 2030. Health Serv. insights 15, 11786329221121214. 10.1177/11786329221121214 36081830 PMC9445529

[B5] Almalki M. Fitzgerald G. Clark M. (2011). Health care system in Saudi Arabia: an overview. East Mediterr. Health J. 17 (10), 784–793. 10.26719/2011.17.10.784 22256414

[B6] Al-Omar H. A. Almuhsin A. A. Almudaiyan L. H. Al-Najjar A. H. Abu Esba L. C. Almodaimegh H. (2025). A strategic framework for synergizing managed entry agreement efforts to access pharmaceutical products in saudi Arabia-results from a multi-stakeholder workshop. J. Med. Econ. 28 (1), 753–765. 10.1080/13696998.2025.2506967 40371839

[B7] Amrein A. C. (2020). Exploring the integration of evidence-based medicine, quality of life considerations, and health economics for rare diseases. Queensland, Australia: University of Southern Queensland.

[B8] Antoñanzas F. Terkola R. Overton P. M. Shalet N. Postma M. (2017). Defining and measuring the affordability of new medicines: a systematic review. Pharmacoeconomics 35 (8), 777–791. 10.1007/s40273-017-0514-4 28477220

[B9] Balkhi B. Almuaither A. Alqahtani S. (2023). Cross-national comparative study of orphan drug policies in Saudi Arabia, the United States, and the european union. Saudi Pharm. J. 31 (9), 101738. 10.1016/j.jsps.2023.101738 37638213 PMC10458326

[B10] Boeira L. Hayter E. Oliver S. Mahlanza-Langer L. Simeon D. Bangpan M. (2025). Efforts towards the institutionalisation of evidence-informed decision-making. BMJ Evidence-Based Med.–112962. 10.1136/bmjebm-2024-112962 PMC7617397 39848631

[B11] Braun V. Clarke V. (2006). Using thematic analysis in psychology. Qual. Res. Psychol. 3, 77–101. 10.1191/1478088706qp063oa

[B12] Chan A. Y. L. Chan V. K. Y. Fan M. Ge M. Pathadka S. Chan E. W. (2020). Access and unmet needs of orphan drugs in 194 countries and 6 areas: a global policy review with content analysis. Value Health 23 (12), 1580–1591. 10.1016/j.jval.2020.06.020 33248513

[B13] Chung C. C. Y. Project H. K. G. Chu A. T. W. Chung B. H. Y. (2022). Rare disease emerging as a global public health priority. Front. Public Health 10, 1028545. 10.3389/fpubh.2022.1028545 36339196 PMC9632971

[B14] Cui Y. Z. Han J. X. (2015). A proposed definition of rare diseases for China: from the perspective of return on investment in new orphan drugs. Orphanet J. Rare Dis. 10, 28. 10.1186/s13023-015-0241-x 25757391 PMC4353681

[B15] Cutillo C. M. Austin C. P. Groft S. C. (2017). A global approach to rare diseases research and orphan products development: the international rare diseases research consortium (IRDiRC). Rare Dis. Epidemiol. Update Overv. 1031, 349–369. 10.1007/978-3-319-67144-4_20 29214582

[B16] Fontrier A.-M. (2022). Market access for medicines treating rare diseases: association between specialised processes for orphan medicines and funding recommendations. Soc. Sci. Med. 306, 115119. 10.1016/j.socscimed.2022.115119 35700552

[B17] GOV.SA (2023). Saudi health council - kingdom of Saudi Arabia. Saudi Government. Riyadh, Saudi Arabia: Digital Government Authority. Available online at: https://www.my.gov.sa/wps/portal/snp/agencies/agencyDetails/AC125/.

[B18] Gupta S. Ai X. Kavuluru R. (2023). Comparison of pipeline, sequence-to-sequence, and GPT models for end-to-end relation extraction: experiments with the rare disease use-case. arXiv. 10.48550/arXiv.2311.13729

[B19] Haendel M. Vasilevsky N. Unni D. Bologa C. Harris N. Rehm H. (2020). How many rare diseases are there? Nat. Rev. drug Discov. 19 (2), 77–78. 10.1038/d41573-019-00180-y 32020066 PMC7771654

[B20] Harris E. (2018). Addressing the needs of Canadians with rare diseases: an evaluation of orphan drug incentives. J. law Biosci. 5 (3), 648–681. 10.1093/jlb/lsy019 31143457 PMC6534751

[B21] Kaywanga F. Alimohamed M. Z. David A. B. Maeda D. Mbarak S. Mavura T. (2022). Rare diseases in Tanzania: a national call for action to address policy and urgent needs of individuals with rare diseases. Orphanet J. Rare Dis. 17 (1), 343. 10.1186/s13023-022-02498-0 36064429 PMC9446714

[B22] Lekander I. N. Ylva S. (2010). Assessing access to innovative treatment: a prevalence-based cost-of-illness study. CiteSeerX.

[B23] Ma N. Nie W. Wang T. Li C. (2013). Current status and countermeasure of the research on rare diseases in China. Life Sci. J. 10 (2), 11–14. 10.9734/IJTDH/2022/v43i241373

[B24] Mak K.-K. Wong Y.-H. Pichika M. R. (2024). Artificial intelligence in drug discovery and development. Drug Discov. Eval. Saf. Pharmacokinet. assays, 1461–1498. 10.1007/978-3-031-35529-5_92

[B25] Mohammadshahi M. Olyaeemanesh A. Ehsani-Chimeh E. Mobinizadeh M. Fakoorfard Z. Akbari Sari A. (2022). Methods and criteria for the assessment of orphan drugs: a scoping review. Int. J. Technol. Assess. Health Care 38 (1), e59. 10.1017/S0266462322000393 35730573

[B26] Nicod E. Annemans L. Bucsics A. Lee A. Upadhyaya S. Facey K. (2019). HTA programme response to the challenges of dealing with orphan medicinal products: process evaluation in selected European countries. Health policy Amsterdam Neth. 123 (2), 140–151. 10.1016/j.healthpol.2017.03.009 28400128

[B27] Program H. S. T. (2021). Health sector transformation program delivery plan 2020-2021. Kingdom of Saudi Arabia: Saudi Arabia: Health Sector Transformation Program.

[B28] Repetto G. M. Rebolledo-Jaramillo B. (2020). Rare diseases: Genomics and public health. Appl. Genomics Public Health, 37–51. 10.1016/b978-0-12-813695-9.00003-0

[B29] Richter T. Nestler-Parr S. Babela R. Khan Z. M. Tesoro T. Molsen E. (2015). Rare disease terminology and Definitions-A systematic global review: report of the ISPOR rare disease special interest group. Value Health 18 (6), 906–914. 10.1016/j.jval.2015.05.008 26409619

[B30] Schieppati A. Henter J.-I. Daina E. Aperia A. (2008). Why rare diseases are an important medical and social issue. Lancet 371 (9629), 2039–2041. 10.1016/S0140-6736(08)60872-7 18555915

[B31] Song R. Hall H. I. Green T. A. Szwarcwald C. L. Pantazis N. (2017). Using CD4 data to estimate HIV incidence, prevalence, and percent of undiagnosed infections in the United States. J. Acquir Immune Defic. Syndr. 74 (1), 3–9. 10.1097/QAI.0000000000001151 27509244

[B32] Straum A. K. (2018). Application of an early HTA framework for determining potential cost-effectiveness and value of a medical device the case of the ably bed.

[B33] Walkowiak D. Domaradzki J. Mozrzymas R. Kałużny Ł. Walkowiak J. (2024). Navigating the unique challenges of caregiving for children with rare diseases: are the care experiences of all caregivers the same? A focus on life-limiting rare diseases. J. Clin. Med. 13 (15), 4510. 10.3390/jcm13154510 39124776 PMC11313382

[B34] Zelei T. Mendola N. D. Elezbawy B. Németh B. Campbell J. D. (2021). Criteria and scoring functions used in multi-criteria decision analysis and value frameworks for the assessment of rare disease therapies: a systematic literature review. Pharmacoecon Open 5, 605–612. 10.1007/s41669-021-00271-w 34003484 PMC8611126

