# Supplementary material for: Shaping national rare diseases definition in Saudi Arabia: outcome from health ecosystem multisectoral workshop
Source: Front Pharmacol. 2025 Jul 17;16:1595967. doi: 10.3389/fphar.2025.1595967 (PMC12312012; doi:10.3389/fphar.2025.1595967)
Supplement: Supplementary file 1 [file Table1.docx]

**Supplementary Table 1:** Qualitative and quantitative criteria definitions

| **Theme** | **Descriptors** | **Definition** |
| --- | --- | --- |
| **Qualitative Theme - RDs** | | |
| **Nature** | **Disease** | Any harmful deviation from the normal structural or functional state of an organism, generally associated with certain signs and symptoms and differing in nature from physical injury. |
|  | **Condition** | A health problem with certain characteristics or symptoms. |
|  | **Disorder** | An abnormal physical or mental condition. |
|  | **Syndrome** | A group of signs and symptoms that occur together and characterize a particular abnormality or condition. |
|  | **Symptom** | A physical or mental problem that a person experiences that may indicate a disease or condition. Symptoms cannot be seen and do not show up on medical tests. Some examples of symptoms are headache, fatigue, nausea, and pain |
|  | **Pathologies** | The study of the essential nature of diseases and especially of the structural and functional changes produced by them. |
|  | **Status** | State, particularly in reference to a morbid condition. |
|  | **Severe** | Very intense or extreme, often used to describe a medical condition that is very dangerous or life-threatening. |
|  | **Chronic** | Something that continues over an extended period of time. A chronic condition is usually long-lasting and does not easily or quickly go away. |
|  | **Serious** | A health condition that carries a high risk of mortality and either negatively impacts a person's daily function or quality of life or excessively strains their caregivers. |
|  | **Intractable** | Not easily relieved or cured. |
|  | **High Complexity** | High risk of complications and/or morbidity or mortality. |
|  | **Heterogeneous Group** | A heterogeneous medical condition or heterogeneous disease is a medical term referring to a medical condition with several etiologies (root causes), such as hepatitis or diabetes. |
|  | **Transformative** | Causing or able to cause an important and lasting change in someone or something. |
| **Disease nature affecting the pt** | **Disable** | Any condition of the body or mind (impairment) that makes it more difficult for the person with the condition to do certain activities (activity limitation) and interact with the world around them (participation restrictions). |
|  | **Life-Limiting condition** | A condition, illness or disease that is progressive and fatal, and the progress of which cannot be reversed by treatment. |
|  | **Life-threatening** | Diseases or conditions where the likelihood of death is high unless the course of the disease is interrupted and diseases or conditions with potentially fatal outcomes, where the endpoint of clinical trial analysis is survival. |
|  | **Substantial cause for early death** | Early death, also called premature death, occurs earlier than the average age of death in a population. |
|  | **Long-Term Treatment** | Medical treatment administered over a long period of time. |
|  | **Debilitating** | Causing serious impairment of strength or ability to function. |
| **Etiology** | **Unknown Etiology** | A medical condition that has an unknown cause or origin. |
|  | **Genetic** | Relating to or determined by the origin, development, or causal antecedents of something. |
|  | **Hereditary** | Genetically transmitted or transmittable from parent to offspring. |
|  | **Partially understood** | Partially means to some extent: in some degree. |
|  | **No satisfactory** | Something that is satisfactory is acceptable to you or fulfils a particular need or purpose. |
|  | **Limited treatment alternative** | Alternative medicine refers to therapeutic approaches taken in place of traditional medicine and used to treat or ameliorate disease. |
|  | **Morbidity** | Refers to having a disease or a symptom of disease, or to the amount of disease within a population. |
|  | **Mortality** | Refers to the state of being mortal (destined to die). |
|  | **Perinatal** | Occurring in, concerned with, or being in the period around the time of birth. |

| **Theme** | **Descriptors** | **Definition** |
| --- | --- | --- |
| **Qualitative Theme - RDs** | | |
| **Disease nature affecting the pt.s Society.** | **Considerable reduction in an individual's quality of life** | Reduction in the standard of health, comfort, and happiness experienced by an individual or group. |
|  | **Considerable reduction in socio- economic potential** | Socioeconomic status (SES) is defined as a measure of one's combined economic and social status and tends to be positively associated with better health. |
| **Population Characteristics** | **Low Prevalence** | The proportion of the population with a condition at a specific point in time (point prevalence) or during a period of time (period prevalence). |
| **Quantitative Theme - RDs** | | |
| **Measurements** | **Prevalence** | Prevalence is the proportion of a population who have a specific characteristic in a given time period. |
|  | **Incidence** | The frequency with which a disease occurs in a population. |
|  | **Frequency** | The number, proportion, or percentage of items in a particular category in a set of data. |
|  | **Threshold** | A level, point, or value above which something is true or will take place and below which it is not or will not |
|  | **Ratio** | The relationship in quantity, amount, or size between two or more things. |
|  | **Estimated measure** | A rough calculation or one based on incomplete data. |
|  | **Range** | the difference between the least and greatest values of an attribute or of the variable of a frequency distribution. |
|  | **Percentage** | A part of a whole expressed in hundredths. |

| **Theme** | **Descriptors** | **Definition** |
| --- | --- | --- |
| **Qualitative Theme - URDs** | | |
| **Population Characteristics** | **Population** | The population of a country or area is all the people who live in it. |
|  | **People** | Human beings making up a group or assembly or linked by a common interest. |
|  | **Persons** | A person is an individual human being. |
|  | **Inhabitants** | The inhabitants of a place are the people who live there. |
| **Nature** | **Disease** | Any harmful deviation from the normal structural or functional state of an organism, generally associated with certain signs and symptoms and differing in nature from physical injury. |
|  | **Chronic** | Something that continues over an extended period of time. A chronic condition is usually long-lasting and does not easily or quickly go away. |
| **Quantitative Theme - URDs** | | |
| **Measurement** | **Prevalence** | Prevalence is the proportion of a population who have a specific characteristic in a given time period. |
|  | **Incidence rate** | The number of new cases within a time period (the numerator) as a proportion of the number of people at risk for the disease (the denominator). |

| **Theme** | **Descriptors** | **Definition** |
| --- | --- | --- |
| **Qualitative Theme - ODs** | | |
| **Indications** | **Diagnosis** | the art or act of identifying a disease from its signs and symptoms. |
|  | **Treatment** | The use of an agent, procedure, or regimen, such as a drug, surgery, or exercise, in an attempt to cure or mitigate a disease, condition, or injury. |
|  | **Prevention** | Action taken to decrease the chance of getting a disease or condition |
|  | **Prophylaxis** | An attempt to prevent disease. |
|  | **Rehabilitation** | A process to restore mental and/or physical abilities lost to injury or disease, in order to function in a normal or near-normal way. |
|  | **Controlling** | When public health experts talk about controlling a disease, they mean reducing the number of new infections, the number of people currently infected, and the number of people who become sick or die from a disease in local settings. |
|  | **Curative** | Relating to or used in the cure of diseases : tending to cure. |
| **Benefits from taking the treatment.** | **Clinical added value** | An assessment of the therapeutic (or diagnostic) progress provided by a medicinal product – notably in terms of efficacy or safety – compared with existing alternatives. It measures the medical added value of the medicine compared with existing therapies. |
| **Population Characteristics** | **Low Prevalence** | Prevalence is the proportion of a population who have a specific characteristic in a given time period. |
| **Quantitative Theme – ODs & UODs** | | |
| **Measurements** | **Prevalence** | Prevalence is the proportion of a population who have a specific characteristic in a given time period. |
|  | **Cost-effectiveness threshold** | Cost-effectiveness thresholds (CETs) are typically used to assess whether an intervention is worthwhile and should reflect health opportunity cost. |
|  | **willingness to pay (WTP) of <3 times gross domestic product (GDP) per capita/QALY** | - WTP refers to the amount of money that individuals would give up in order to avoid one episode of the health endpoint before it has occurred.  - GDP refers to the gross national product excluding the value of net income earned abroad.  - QALY refers to the measurement of the state of health of a person or group in which the benefits, in terms of length of life, are adjusted to reflect the quality of life. One quality-adjusted life year (QALY) is equal to 1 year of life in perfect health. |

| **Theme** | **Descriptors** | **Definition** |
| --- | --- | --- |
| **Qualitative Theme - UODs** | | |
| **Nature** | **Medicines** | A substance or preparation used in treating disease. |
|  | **Drug** | A substance used in the diagnosis, treatment, or prevention of a disease or medical condition, including prescription drugs, over-the-counter drugs, and illegal drugs. |
|  | **Diseases** | Any harmful deviation from the normal structural or functional state of an organism, generally associated with certain signs and symptoms and differing in nature from physical injury. |
|  | **Conditions** | A health problem with certain characteristics or symptoms. |
| **Indication** | **Indications** | A sign, symptom, or medical condition that leads to the recommendation of a treatment, test, or procedure. |
|  | **Treat** | The use of an agent, procedure, or regimen, such as a drug, surgery, or exercise, in an attempt to cure or mitigate a disease, condition, or injury. |
| **Population Characteristics** | **Patients** | A person who is receiving medical treatment from a doctor or hospital. A patient is also someone who is registered with a particular doctor. |
|  | **Persons** | A person is an individual human being. |
|  | **People** | Human beings making up a group or assembly or linked by a common interest. |
