# Supplementary material for: Shaping national rare diseases definition in Saudi Arabia: outcome from health ecosystem multisectoral workshop
Source: Front Pharmacol. 2025 Jul 17;16:1595967. doi: 10.3389/fphar.2025.1595967 (PMC12312012; doi:10.3389/fphar.2025.1595967)
Supplement: Supplementary file 2 [file Table2.docx]

**The Supplementary Table 2** RD Qualitative Criteria

| **Nature** | **Etiology** | **Disease nature affecting the pt** | **Unmet need** | **Disease nature affecting the pt’s Society** | **Population Characteristics** |
| --- | --- | --- | --- | --- | --- |
| Disease | Unknown Etiology | Disable | Lack of Resources | Considerable reduction in an individual's quality of life | Low Prevalence |
| Condition | Genetic | Life-Limiting condition | No satisfactory | Considerable reduction in socio- economic potential | Small % of the Population |
| Disorder | Hereditary | Life-threatening | Paucity of treatment availability |  | Low Occurrence |
| Syndrome / Symptom | Partially understood | Substantial cause for early death |  |  | Rarely afflict the population |
| Pathologies |  | Long-Term Treatment | Investment to develop new treatments |  |  |
| Status |  | Debilitating |  |  |  |
| Severe |  |  | Limited treatment alternative |  |  |
| Chronic |  |  | Difficult to Justify the development risk |  |  |
| Serious |  |  |  |  |  |
| Intractable |  |  | Combined efforts to prevent significant morbidity, prevent early mortality |  |  |
| High Complexity |  |  |  |  |  |
| Heterogeneous Group |  |  |  |  |  |
| Transformative |  |  |  |  |  |
