# Supplementary material for: Shaping national rare diseases definition in Saudi Arabia: outcome from health ecosystem multisectoral workshop
Source: Front Pharmacol. 2025 Jul 17;16:1595967. doi: 10.3389/fphar.2025.1595967 (PMC12312012; doi:10.3389/fphar.2025.1595967)
Supplement: Supplementary file 3 [file Table3.docx]

**Supplementary Table 3:** The Vevox-implemented questions

**Ice break Questions**

1. Are you a coffee person or a tea person?
2. **Coffee**
3. **Tea**
4. If you could have one of these superpowers, which one would you choose?
5. **Be invisible whenever you want**
6. **Superhuman Strength**
7. **Talk to animals**
8. **Read minds**
9. **Be able to fly**
10. If you could travel in time, what would you want to see?
11. **The past**
12. **The future**
13. **There is enough in the present**
14. How are you feeling?
15. **Happy**
16. **Confused**
17. **Sleepy**
18. **Motivated**

**Demographics and professional history of workshop participants.**

1. Gender
2. **Male**
3. **Female**
4. Age (years)
5. **25–34**
6. **35–44**
7. **45–54**
8. **≥ 55**
9. What is your educational background?
10. **Physician**
11. **Pharmacist**
12. **Nurse**
13. **Economist**
14. **Finance**
15. **Other**
16. What is your current professional background? (you can choose more than one option)
17. **Clinical Pharmacist**
18. **Chair/member of pharmacy and therapeutic committees (PTCs)**
19. **Academic professor**
20. **Policy maker**
21. **Key opinion leader (in a specific therapy area)**
22. **Payer**
23. **Regulator**
24. **Researcher/member of research agencies**
25. **Clinical guideline expert**
26. **Health authority official**
27. **Physician**
28. **Medical insurance**
29. **Other**
30. What is the nature of your workplace setting?
31. **Government**
32. **Private**
33. **Authority**
34. **Other**

**Introductory**

1. What’s your level of understanding of ODs accessibility in Saudi Arabia?
2. **I’m completely green**
3. **I have some basic knowledge**
4. **I have solid background**
5. **I’m an expert**
6. What criteria help you decide whether to say “no” to something or commit to it?

------------------------------------------------------------------------------------------------------------

**National Rare Disease (RD) Definition - Proposal**

1. Do you think the comprehensive National RD definition should include?
2. **Qualitative Descriptor**
3. **Quantitative Descriptor**
4. **Both**
5. Which qualitative descriptor do you prefer most to be included in the National RD definition? You can select more than one option
6. **Disease**
7. **Condition**
8. **Disorder**
9. **Syndrome**
10. **Symptom**
11. **Pathologies**
12. **Status**
13. **Severe**
14. **Chronic**
15. **Serious**
16. **Intractable**
17. **High Complexity**
18. **Heterogeneous Group**
19. **Transformative**
20. Which qualitative descriptor do you prefer most to be included in the National RD definition? You can select more than one option
21. **Unknown Etiology**
22. **Genetic**
23. **Hereditary**
24. **Partially understood.**
25. Which qualitative descriptor do you prefer most to be included in the National RD definition? You can select more than one option
26. **Disable**
27. **Life-Limiting condition**
28. **Life-threatening**
29. **Substantial cause for early death**
30. **Long-Term Treatment**
31. **Debilitating**
32. Which qualitative descriptor do you prefer most to be included in the National RD definition? You can select more than one option
33. **Lack of Resources (Require Specialized Medical Care, Difficulties in Obtaining Timely Accurate Diagnoses)**
34. **No satisfactory**
35. **Paucity of treatment availability**
36. **Investment to develop new treatments.**
37. **Limited treatment alternative**
38. **Difficult to Justify the development risk.**
39. **Combined efforts to prevent significant morbidity.**
40. **Combined efforts to prevent early mortality.**
41. **Combined efforts to prevent perinatal.**
42. Which qualitative descriptor do you prefer most to be included in the National RD definition? You can select more than one option
43. **Considerable reduction in an individual's quality of life**
44. **Considerable reduction in socio- economic potential**
45. Which qualitative descriptor do you prefer most to be included in the National RD definition? You can select more than one option
46. **Low Prevalence**
47. **Small % of the Population**
48. **Low Occurrence**
49. **Rarely afflict the population**
50. Would you like to include a descriptors that reflect the population size, like, low, nearly or cut off clear number?
51. **Yes**
52. **No**
53. Why?

------------------------------------------------------------------------------------------------------------

1. Which **quantitative descriptor** do you prefer most to be included in the National RD definition? You can select more than one option
2. **Prevalence**
3. **Absolute # of patients**
4. **Incidence**
5. **Frequency**
6. **Number of cases reference Threshold**
7. **Ratio**
8. **Estimated measure**
9. **Range**
10. **Percentage**
